# Emergency Department Time Course for Mild Traumatic Brain Injury Workup

**DOI:** 10.5811/westjem.2018.5.37293

**Published:** 2018-06-13

**Authors:** Edward A. Michelson, J. Stephen Huff, Mae Loparo, Rosanne S. Naunheim, Andrew Perron, Martha Rahm, David W. Smith, Joseph A. Stone, Ariel Berger

**Affiliations:** * Paul L. Foster School of Medicine, Texas Tech University Health Sciences Center, Department of Emergency Medicine, El Paso, Texas; † University of Virginia Health System, Department of Emergency Medicine, Charlottesville, Virginia; ‡ University Hospitals Cleveland Medical Center, Department of Emergency Medicine, Cleveland, Ohio; § Washington University School of Medicine, Department of Emergency Medicine, St. Louis, Missouri; ¶ Maine Medical Center, Department of Emergency Medicine, Portland, Maine; || Barnes Jewish Hospital, Department of Emergency Medicine, St. Louis, Missouri; # Integris Baptist Medical Center, Department of Emergency Medicine, Oklahoma City, Oklahoma; ** Brody School of Medicine, East Carolina School of Medicine, Department of Emergency Medicine, Greenville, North Carolina; †† Evidera, Bethesda, Maryland

## Abstract

**Introduction:**

Mild traumatic brain injury (mTBI) is a common cause for visits to the emergency department (ED). The actual time required for an ED workup of a patient with mTBI in the United States is not well known. National emergency medicine organizations have recommended reducing unnecessary testing, including head computed tomography (CT) for these patients.^10^

**Methods:**

To examine this issue, we developed a care map that included each step of evaluation of mTBI (Glasgow Coma Scale Score 13–15) – from initial presentation to the ED to discharge. Time spent at each step was estimated by a panel of United States emergency physicians and nurses. We subsequently validated time estimates using retrospectively collected, real-time data at two EDs. Length of stay (LOS) time differences between admission and discharged patients were calculated for patients being evaluated for mTBI.

**Results:**

Evaluation for mTBI was estimated at 401 minutes (6.6 hours) in EDs. Time related to head CT comprised about one-half of the total LOS. Real-time data from two sites corroborated the estimate of median time difference between ED admission and discharge, at 6.3 hours for mTBI.

**Conclusion:**

Limiting use of head CT as part of the workup of mTBI to more serious cases may reduce time spent in the ED and potentially improve overall ED throughput.

## INTRODUCTION

According to the United States (U.S.) Centers for Disease Control and Prevention, the incidence of traumatic brain injury (TBI) has increased by nearly 60% from calendar-year (CY) 2001 to CY2010 (from 521 per 100,000 persons to 824 per 100,000 persons).^1^ Visits to the emergency department (ED) resulting in a diagnosis of TBI increased by 29.1% (95% confidence interval [CI], 18.9%–39.2%) in the time period 2006 to 2010, whereas the total number of ED visits increased by only 3.6% (95% CI [−0.7%–8.0%]) during the same period.^2^ A recent analysis suggests that nearly five million patients present to U.S. EDs annually to be evaluated for head injury, and that approximately one-half of them are diagnosed with a TBI.^3^ Further, most patients who present to the ED with suspected TBI have mild TBI (mTBI), estimated to be as high as 94.5%.^4^,^5^

In addition to obtaining a detailed patient history and thorough physical examination, computed tomography (CT) head imaging has frequently been part of the diagnostic workup, and has been recommended for most if not all patients with suspected mTBI.^4^–^6^ CTs are now typically performed on >80% of patients who present to the ED with suspected TBI.^7^ However, there has been growing concern about the radiation exposure and cost associated with CT. The decision to obtain a head CT also adds time to the ED visit (primarily waiting for the scan to be run and/or read), and requires additional resources, including use of the CT scanner and additional hospital staff. In a study by Rogg and colleagues, 8,312 ED patients who received a head CT reported a median time of 3 hours and 13 minutes (193 minutes) between the patient arrival and the CT preliminary report in high-volume EDs.^8^ They concluded that head CT has a significant impact on patient wait times.

The present article explores the workflow and associated time of assessing a patient with a head injury. We evaluated the process by constructing a detailed, theoretical care map, retrospectively testing the care map against actual patient time data and comparing the results to those published in the literature. Using such a detailed care map from admission to discharge from the ED could help identify specific steps in care, which could significantly decrease patient wait times. The purpose of this study was to understand times associated with all of the steps in ED workup of a patient with mTBI, from the point of initial ED presentation to discharge. An understanding of each step in the workup and associated times is necessary to identify opportunities to shorten the total workup of these patients.

## METHODS

We developed a theoretical care map describing the steps in the typical workup of a patient presenting to the ED following a mTBI. The care map was based on a facilitated consensus panel discussion between three experienced, academic, board-certified emergency physicians, each with at least 20 years of experience at academic, high-volume EDs, (JH, EM, RN,) during a four-hour meeting and two rounds of follow-up comments on the care map to gain consensus. The working draft was then presented for review to a larger expert panel of experienced emergency medicine nurses, nurse practitioners and physicians from around the U.S. (AP, DS, EM, JS, ML, MR, RN, SH). The care map was further revised based on feedback from the second group of reviewers, and subsequently finalized.

**Population Health Research Capsule**

What do we already know about this issue?

Traumatic brain injury (TBI) is a common cause for an emergency department (ED) visit. National emergency medicine organizations have recommended reducing unnecessary testing, including head computed tomography (CT) for these patients.

What was the research question?

What are the times associated with all the steps in ED workup of a patient with mild TBI, from the point of initial ED presentation to discharge?

What was the major finding of the study?

Evaluation for mild TBI in the ED was estimated at 401 minutes (6.6 hours) in EDs. Time related to head CT comprised about one-half of the total length of stay.

How does this improve population health?

Limiting use of head CT as part of the workup of mild TBI to more serious cases may reduce time spent in the ED and potentially improve overall ED throughput.

The care map of workup for a ED visit for suspected mTBI included 10 unique steps identified during a “typical” episode of care, beginning with initial presentation to the ED and ending with discharge (Figure). For each of these steps, estimates of time required to perform the step were identified and discussed with both consensus panels. The larger expert panel confirmed the steps in the care map. The figure summarizes the care map of 10 steps associated with ED visit for suspected TBI. The care map demonstrates a range of work/time flow differences. However, the map was only tested in sites with similar high volume, to compare with the Rogg published data, and also to validate the steps and timing contained in the care map.^8^

In the second part of the study, we tested the assumptions in the theoretical care map using retrospective data from EDs at two major U.S. teaching hospitals. Both were high-volume EDs, as defined above, one seeing 60,000 patients annually with four CT scanners and the second seeing 70,000 patients annually with two CT scanners. We collected retrospective observational data on ED length of stay (LOS) defined as the time between registration and ED discharge. This data was extracted from the electronic data collection form from two of the 11 sites from a larger published clinical trial.^9^ All patients were between the ages of 18–85 (mean 45.7, standard deviation [SD]=19.8), 50% male, with Glasgow Coma Score between13–15 (mean 14.9), and were evaluated within 72 hours of injury (mean 12.7 hours, SD=.47). The time of admission and discharge were obtained from the study electronic data capture form and confirmed from the electronic health record. From this data we calculated time interval between admission and discharge from the ED. Median LOS was calculated, as well as first quartile and third quartile, and these values were compared with the Rogg study data.^8^ We conducted analyses using Microsoft Excel 2016 MSO (16.0.8431.2046) 32-bit (Microsoft Corporation, Redmond, Washington).

## RESULTS

In the theoretical care map, total and component time of mTBI evaluation in U.S. EDs is summarized in Table 1. We estimated LOS as 401 minutes (~6.6 hours) in the EDs.

Step 2 of the care map outlines the triage of the patient, first by a registered nurse, and then by a provider. Many EDs, but not all, now use a provider in triage model to do brief patient assessments “up front” and initiate testing before the patient is placed in a room (Step 3). Time spent between waiting for transport to the CT unit (Step 4) and physician reassessment following radiologist review of the CT (Step 9) was estimated to be 151 minutes (~2.5 hours) at EDs. The difference is due to delays in transport, as well as longer times for radiology reads in high-volume sites. Regardless of hospital volume, the acquisition of the CT itself was estimated to take only 12 minutes (3.4% of LOS at hospitals).

Actual retrospective data from the two study hospitals showed a mean LOS of 7.9 hours ± 7.0 hours. The comparison between the present study (N=125) and the Rogg 8 study (N=8,312) LOS median, first quartile, and the third quartile are presented in Table 2. The Rogg study^8^ included both CT+ and CT− subjects in its population but did not separate the population into CT+ and CT− subgroups. In the present study, we present the data for combined CT groups, as Rogg and colleagues, and in addition, we split the study population into CT+ and CT− patients to examine length of time for the two subtypes of patients. There was no significant difference in LOS between the CT+ and CT− patients (p=0.8) in the present study. Furthermore, it was of interest to note that there was no difference in the CT− and CT+ data.

## DISCUSSION

The purpose of this study was to understand times associated with all of the steps in ED workup of a patient with mTBI, from the point of initial ED presentation to discharge. An understanding of each step in the workup and associated times is necessary to identify opportunities to shorten the total workup of these patients. We created a care map using input from eight healthcare professionals with expertise in emergency care. The care map developed comprised a total of 10 steps (Figure). The results from the 125 subjects, retrospective study LOS closely compare with the over 8,000-subject Rogg study^8^ (6.3 hours, and 6.4 hours respectively).

Despite recent recommendations to the contrary,^11^ CTs of the head continue to be frequently ordered as part of the workup of suspected mTBI. While CT imaging identifies problems that otherwise may be missed by physical examination (e.g., fractures, epidural and subdural bleeds, and subarachnoid hemorrhage), such scans are “positive” for only 6%–8% of patients with mTBI, and <1% of patients with mTBI are found to require neurologic intervention.^11^,^12^ Inclusion of CT adds substantially to the total workup of mTBI. Moreover, CT is costly to the patient, does not establish or confirm diagnosis of concussion and increases – albeit nominally – exposure to radiation and subsequent potential risk of cancer. Furthermore, given its limitations a negative CT does not exclude significant TBI with associated symptoms. The American College of Emergency Physicians “Choosing Wisely” guidelines^10^ for emergency medicine were designed to avoid unnecessary testing. The first recommendation (of 10) was to “Avoid computed tomography (CT) scans of the head in emergency department patients with minor head injury who are at low risk based on validated decision rules.”^10^ In addition to reducing patient exposure to radiation, results of our study suggest that avoiding unnecessary CTs could substantially reduce time spent to render care. Specifically, elimination of the head CT and all related steps (i.e., steps 4–9), would result in an estimated time savings of 151 minutes, as a substantial proportion of the time required to assess suspected mTBI was attributable to steps following the decision to order a CT. The removal of these steps may require additional physician time to discuss benefits of avoiding a CT. While this represents a “best-case” estimate in that it assumes that no patient would undergo a CT, it should be noted that a recent report found that 82% of ED patients with suspected TBI underwent CT, producing about 3.9 million head scans annually; 91% of these scans (3.5 million) were negative.^3^ Moreover, <1% of patients with mTBI require neurological intervention.^10^,^11^ There is a need for an alternative, objective triage tool or decision rule that could potentially aid in the safe reduction of the number of CTs ordered. Moreover, when working up a patient with mTBI a normal CT does not rule out the presence of a functional brain injury or concussion.

The U.S. government, and consequently hospitals, have become increasingly concerned about long wait times in the ED and patient satisfaction. Median time (in minutes) from ED arrival to departure is a quality-of-care metric developed by the U.S. Centers for Medicare and Medicaid Services used by the government to determine accreditation, external oversight, external and internal quality improvement, pay-for-reporting, and public reporting.^12^ Reducing the time from presentation to diagnosis by limiting CT or other recognized inefficiencies from the care map could contribute to increased levels of patient satisfaction.

## LIMITATIONS

The present study has some limitations. First, the care map was not based on time-and-motion studies of actual EDs, but rather on the input of a panel of experienced emergency medicine providers. Second, while assumptions are required to develop care maps, the ones made herein were designed to describe the course of care for the typical patient who presents to the ED with suspected mTBI. Our care map estimated times associated with workup to the point of a decision for admission or discharge. We did not study the further times or steps that patients admitted following workup might experience. Accordingly, the degree to which findings from this study are generalizable to the entire relevant patient population is conjectural.

Third, we did not attempt to study the total charges associated with the workup of mTBI. Future work could examine the costs to insurers as well as patients associated with use of the ED, professional fees from emergency physicians as well as the professional and technical fees associated with a head CT. Finally, the potential impact of more selective utilization of head CT and subsequent quicker disposition of patients with mTBI on overall ED throughput was not studied. New point-of-care technologies, now available for diagnosing mTBI, might enhance practitioner confidence for more selective use of head CT as well.^9^

## CONCLUSION

We found that approximately one-half of the time associated with the current typical ED evaluation work-up of suspected mTBI is the result of the decision to order and the time and resources necessary to complete and obtain an interpretation of a head CT. Given the large number of visits for suspected mTBI, any strategies that result in more selective utilization of head CTs may reduce the time and cost required to render care.

## Figures and Tables

**Figure:**
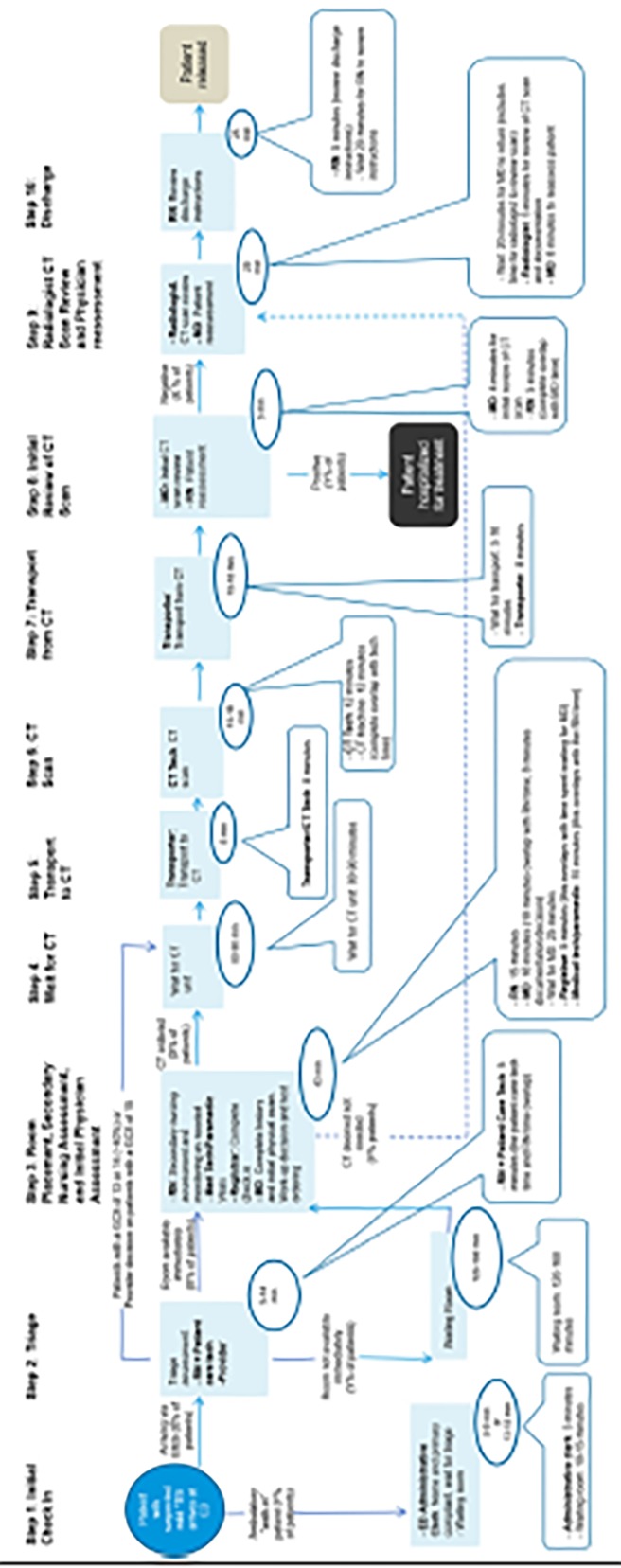
Care map of 10 steps associated with emergency department (ED) visit for suspected traumatic brain injury. *RN*, registered nurse; *CT*, computed tomography; *GCS*, glasgow coma scale; *TBI*, traumatic brain injury. *As defined by either New Orleans criteria or Canadian CT criteria.

**Table 1:** Total estimated time associated with diagnosis of mild traumatic brain injury in United States emergency departments.

Step	Time
1. Initial check in	16 minutes
2. Triage	161 minutes
2a. RN triage assessment	6 minutes
2b. Provider triage assessment*	5 minutes
2c. Waiting room	150 minutes
3. Room placement, secondary nursing assessment, and initial physician assessment	48 minutes
4. Wait for CT	85 minutes
5. Transport to CT	8 minutes
6. CT scan	12 minutes
7. Transport from CT and radiologist CT review	13 minutes
8. Return to ED, CT review, and nursing reassessment	5 minutes
9. Physician reassessment	28 minutes
10. Discharge	25 minutes
Total	401 minutes (6.6 hours)

*ED*, emergency department; *CT*, computed tomography; *RN*, registered nurse.

**Table 2:** Length of stay comparison between the Rogg et al. study and the present study.

Length of stay (hours)	Rogg patients	Present study patients
Median	6.4	6.3
Quartile 1	4.6	3.8
Quartile 3	9	8.8
